# Supplementary material for: The whole day matters after stroke: Study protocol for a randomized controlled trial investigating the effect of a ‘sit less, move more, sleep better’ program early after stroke
Source: PLoS One. 2023 Dec 7;18(12):e0290515. doi: 10.1371/journal.pone.0290515 (PMC10703225; doi:10.1371/journal.pone.0290515)
Supplement: S1 File — (PDF) [file pone.0290515.s002.pdf]

The whole day matters after stroke: Moving towards precision rehabilitation guided by behavioural and imaging markers

Project summary

Background

Prolonged sedentary behaviour is associated with worse functional outcomes poststroke.[1] ***The effect of reducing sedentary behaviour early after stroke remains unknown.*** Leukoaraiosis or cerebral white matter disease, recognizable on magnetic resonance imaging (MRI) as areas of hyperintensities,[2] has gained significance as a potential moderator of stroke recovery.[3,4] ***No specific rehabilitation intervention has been developed for survivors of stroke with leukoaraiosis.***

The **goals** of this research project are to:

1. Test the effect of reducing sedentary behaviour early after stroke on functional mobility and global disability outcomes.
2. Determine the impact of leukoaraiosis on response to poststroke rehabilitation.
3. Explore the associations of accelerometry metrics with functional mobility and global disability outcomes poststroke.

Methods

We aim to recruit 50 participants, δ7 days of ischemic stroke onset, aged ≥ 18 years, medically stable as deemed by physicians, able to walk at least 5 meters with/without gait aid, and ongoing walking goals (walk speed <1.0 meter/second). Demographic and stroke characteristics, including stroke risk factors, infarct location and volume, leukoaraiosis on MRI, and acute stroke treatments (e.g. thrombectomy) will be determined and documented. Subsequently, participants will be set up to wear activPAL accelerometer, validated in stroke,[5] for one week. Following randomization, a behaviour change intervention will span 6 weeks with final follow-up assessments at 90 days.

Primary outcomes: modified Rankin Scale and Timed-Up and Go (TUG) score.

Plan for Data Analysis

Compositional data analysis and generalized estimating equations with R software will be used to model the effect of reducing sedentary behaviour and impact of leukoaraiosis on response to rehabilitation. The correlation, responsiveness, and predictive value of the activPAL outcomes as adjunct to the mRS and TUG measures will be evaluated using machine learning, logistic regression, and receiver operating curves.

Significance and Expected Results

The first 90 days after stroke represents a critical window of neuroplasticity. Frequent interruptions in sedentary behaviour, using a whole-day approach, may improve function and recovery, especially for poor responders. If we find this rehabilitation approach to be effective for survivors of stroke with leukoaraiosis, then it could be useful for improving decision-making. Accelerometry as an adjunct to the MRS will increase the granularity of outcome measurement poststroke.

Background/Rationale

The main aim of rehabilitation is to enhance recovery trajectories and improve adaptation to loss of neurologic function with a view to promote function and prevent stroke recurrence.[6] Rehabilitation outcomes after stroke are variable, while some patients respond well to rehabilitation, others do not.[7] Stroke is a heterogeneous disorder with varying brain and behavioural changes across survivors, which poses a significant challenge in using a "one-size-fits-all" approach to rehabilitation.[8] Clinician impression, age, and stroke severity are important considerations for determining "rehabilitation potential",[9] although using "rehabilitation potential" to limit access to rehabilitation is currently being challenged.[10] Integrating the knowledge around mechanisms of stroke recovery, biomarkers, and development of targeted interventions to inform precision rehabilitation is a main challenge in rehabilitation science. Rehabilitation interventions would need to appreciate the heterogeneity in stroke to ensure that the right patient receives the right intervention at the right time point with the right outcome measurement that is responsive to change.[11] Motor recovery through neuroplasticity occurs mostly in the early period after stroke and tends to plateau by 3-6 months.[12,13] Recent evidence suggests that the window of critical neuroplasticity might be within the first few weeks, up to 90 days after stroke onset, and optimized rehabilitation during this critical window of recovery is important.[14,15] Preclinical animal studies have shown that higher number of repetitions is associated with better functional recovery after stroke, with upwards of 600-700 repetitions per day required in those with the most severe impairments.[16] ***The optimal dose of rehabilitation in humans remains unknown. A whole-day approach to optimize stepping time and repetitions through frequent interruptions in sedentary time may be beneficial.***[17]

Understanding the individual-level factors such as novel imaging biomarkers,[3,18] may help to distinguish potential responders from non-responders to stroke rehabilitation to guide clinical decision making. Leukoaraiosis or cerebral white matter disease, recognizable on magnetic resonance imaging (MRI) as areas of hyperintensities,[2] has gained significance as a potential moderator of poststroke recovery.[3,4] The pathogenesis of leukoaraiosis is unclear, however, they may represent the brain's endpoint of poorly controlled cardiometabolic risk factors such as diabetes,[19] hypertension,[20] or smoking.[21] In older adults, leukoaraiosis is associated with impairments in gait parameters, including step length and cadence, possibly due to ongoing axonal changes.[22,23] After a stroke, the impact of leukoaraiosis become more evident,[24] possibly due to a diminished capacity of the cerebral tissue to respond to the brain insult.[25] For example, following mechanical thrombectomy poststroke, only 24% of patients with severe leukoaraiosis had "good" 90-day mRS score compared to 85% of patients without leukoaraoisis.[3] Although the extent of acute infarct volume is associated with motor recovery, this association is distorted by the severity of leukoaraiosis.[4] To date, there are no known therapies to slow down the progression of leukoaraiosis and no rehabilitation interventions have been specifically developed for survivors of stroke with leukoaraiosis. ***Using leukoaraiosis to stratify survivors of***

stroke may potentially help to triage patients who require a different approach to rehabilitation that uses the whole day to promote activity while reducing sedentary behaviour.

The modified Rankin Scale (mRS) is a well-established outcome measure of global disability and endpoint outcome scale in stroke studies with outcomes dichotomized as good [mRS grade 0-2] or poor [mRS grade ≥3].[26,27] However, the mRS has been critiqued for not sufficiently detecting clinically important changes in impairment, activities of daily living, or mobility.[26-28] Moreover, there is individual variability in motor recovery and function after stroke, even among people with similar initial motor impairment.[29] The mRS alone as a clinical endpoint measure after stroke may provide incomplete information on recovery and function. Wearable sensors hold great promise for measurement and monitoring of mobility and activity behaviour poststroke. Understanding the whole picture of how well a stroke survivor with/without leukoaraiosis performs both during and outside structured therapy may provide critical information that can be used to adapt and progress therapy and rehabilitation interventions. Wearable activity sensors allow for unsupervised, continuous, and objective monitoring of performance during and outside structured therapy. The timestamped recordings of exercise and non-exercise tasks over the whole day provides a representative and reliable account of performance. Whole day movement behaviours (sleep, physical activity, and sedentary time) can provide useful information since movement behaviours are co-dependent.[30] Increasing time spent in one behaviour displaces time in the other behaviours within a 24-hour period.[30] There is growing interest in studying the effects of combinations of movement behaviours.[31] For example, extra time in physical activity must come from either sleep or sedentary behaviour or both. Physical activity, sleep and sedentary behaviour are associated with health outcomes.[32,33]***Accelerometry as an adjunct to the mRS may lead to increased granularity of measurement to detect changes in mobility and activity behaviour that may enhance clinical decision-making***. Using novel compositional data analysis developed for co-dependent data,[30,32] this proposed project would provide preliminary data to explore the associations between movement behaviours composition, impact of leukoaraiosis, and response to rehabilitation.

Feasibility

We conducted a feasibility study (n=34), that utilized wearable sensors (research-grade activPAL) for measurement and consumer physical activity monitor for self-monitoring, to enhance self-efficacy to “sit less and move more” after stroke.[34] Within one month of discharge from inpatient stroke rehabilitation, participants underwent 8-weeks sedentary behaviour change intervention.[34] The program was feasible and well-received by survivors of stroke.[35] Relative to baseline, we found that sedentary time decreased by 54 minutes per day (SD: 13.8 minutes, p<0.01) after the intervention. Other findings, that have not yet been published, suggest that after adjusting for age, sex, and cognition, *moving more* by 50 minutes per day relative to other whole-day behaviours (lying/sitting/standing) was associated with a predicted change in TUG score of -3.8 seconds (95% CI: -6.1 to -1.4; **see Figure 1**). *Through collaboration with stroke*

neurologists/co-investigators at the University of Alberta Hospital (Dr. Glen Jickling and Dr. Brian Buck), leukoaraiosis will be determined from MRI scans and further analyzed.

As with any new intervention introduced to standard of care, the research team will put into place processes to ensure feasibility of the whole-day rehabilitation approach early after stroke (e.g., local champion, onboarding resources) facilitated by the materials and resources that will be provided by our research team.

Hypothesis: We hypothesize that a whole-day rehabilitation approach will improve functional mobility and global disability outcomes, moderated by the extent of leukoaraiosis.

Research approach and methods:

Project design: Fifty patients with stroke will be randomized to an experimental (sedentary behaviour change + usual care; n=25) or usual care group (n=25) using computer-generated permuted random block sizes of 2 or 4, stratified by presence of leukoaraiosis. Assessors will be masked to group assignment.

Inclusion criteria: Within 7 days of ischemic stroke onset, aged ≥ 18 years, medically stable as deemed by physicians, able to walk at least 5 meters with/without gait aid, ongoing walking goals (walk speed <1.0 meter/sec).

Exclusion criteria: Have another neurological condition such as multiple sclerosis or Parkinson's disease, or active cancer; uncontrolled high blood pressure or an unstable cardiovascular condition; unable to understand or follow instructions.

Procedure: After informed consent, demographic, and stroke characteristics, including stroke risk factors, infarct location and volume, and acute stroke treatments received (e.g. thrombectomy) will be determined and documented. Consent will be required to grant the study team access to already obtained stroke brain imaging scans (MRI scans) or to obtain new brain imaging (new MRI scan) if no prior MRI has been done since stroke onset. Subsequently, participants will be set up to wear the activPAL for one week, validated in stroke and that accurately distinguishes sedentary from non-sedentary behaviours.[5] The activPAL will be worn on the less affected thigh, separated from the skin by a soft cotton pad. The sedentary behaviour change intervention will span 6 weeks and a final follow-up assessment at 90 days.

Usual care: The control group program will consist of usual inpatient care including therapeutic mobilization by the physical therapy team and general mobilization, as tolerated, by the nursing team.

Experimental (*Behaviour- & Imaging-Guided Stepping Training Early Post-Stroke (BIG STEPS)* intervention: Additional to usual care at the University of Alberta Hospital, the experimental arm will undergo a theory-based behaviour change intervention to improve stepping time relative to reducing sedentary behaviour.[35] Using baseline accelerometry data, personalized goals of replacing sedentary time with

stepping time will be developed. Behaviour-change coaching sessions, outside of structured therapy, will be conducted in person or virtually (where applicable), for up to 1 hour/day, 5 days/week for 6 weeks to enhance self-efficacy to “sit less and move more”. A Fitbit step counter,[36] used as a motivational tool, will provide daily feedback to participants and track adherence. If a participant is discharged from the hospital before 6 weeks, we will complete the follow-up visits at the discharge location or virtually (where applicable). The devices used in this study are licensed for use in Canada by Health Canada.

Study Measures: The primary outcomes at 6 and 12 weeks will be global disability using the mRS score [grade 0-2 vs ≥3][26,27] and Timed-Up and Go (TUG) test to evaluate functional mobility.[37] Secondary outcomes will include time spent stepping and sedentary,[34,38] 10-meter walk test of gait speed,[39] 6-minute walk test of endurance,[40,41] Montreal Cognitive Assessment (MoCA) of cognition,[42] and EQ-5D to assess quality of life.[43,44] National Institute of Health Stroke Scale will classify stroke severity.[40] Leukoaraiosis from MRI scans will be analyzed using standardized automated segmentation protocol of fluid-attenuated inversion recovery sequences, independent of demographic or clinical data.[45]

Timeline: The project is estimated to be completed in 24 months. Ethics/operational approvals are in process with recruitment commencing once approvals are in place. We anticipate recruiting 50 participants from the University of Alberta Hospital within 15 months, with an additional 3 months for follow-up. Data cleaning/processing will be completed after each follow-up period with final data analysis completed within 3-months of the last follow-up. Preparations for major funding submissions and knowledge translation activities will primarily occur in the last quarter of the second year.

Plan for Data Analysis: Compositional data analysis and generalized estimating equations (GEE) with R software will be used to model the effect of reducing sedentary behaviour and the impact of leukoaraiosis on response to rehabilitation. We will use intention-to-treat analysis for primary analysis, with a per-protocol analysis for secondary analysis. The correlation, responsiveness, and predictive value of the activPAL outcomes as adjunct to the mRS and TUG measures will be evaluated with an unsupervised algorithm using Hidden Markov Models (a machine learning approach to classify accelerometry data),[46,47] logistic regression, and receiver operating curves.

Sample size: With assumed difference of 3.8 seconds on the TUG test,[34] a sample of 45 participants, will achieve a power of 0.81 in repeated measures using GEE at 0.05 significance. With an anticipated 10% attrition, 50 participants will be recruited.

Potential for impact

An international group of stroke researchers, the Stroke Recovery and Rehabilitation Roundtable,[48] developed new standards for stroke recovery research. A main part of the standards includes using biomarkers of stroke recovery to stratify people who may respond well to rehabilitation from those that may not to guide clinical trials, advance

our understanding of therapy mechanism, and to predict outcomes.[48] Evaluating the recovery trajectory of survivors of stroke with leukoaraiosis and the effect of early targeted rehabilitation intervention during the critical window of neuroplasticity will advance our understanding of stroke recovery.

Despite rapid advances in acute stroke care, stroke remains the leading cause of adult disability globally.[49] It is concerning that survivors of stroke spend most of their time in sedentary behaviours,[50] including during stroke rehabilitation.[51] Responsible factors may include poor patient compliance with assigned tasks outside of structured therapy or limited dose of rehabilitation after stroke. A meta-analysis showed that each additional hour of sedentary time, beyond 11 hours per day, increases the risk of stroke by 21% in the general population.[52] The influence of sedentary behaviour on stroke recurrence is unknown. The field of stroke recovery, rehabilitation, and restorative neuroscience is still young, and this project will contribute to our understanding of the effect of a whole-day approach to rehabilitation early poststroke, and the specific effect on survivors of stroke with leukoaraiosis. Using a combination of behavioural and imaging markers may help to better understand and implement targeted rehabilitation early after stroke to optimize function and recovery.

The outcome of this work will contribute to better poststroke rehabilitation and ultimately reduce the debilitating effects of strokes. Results may improve decision making for inpatient stroke rehabilitation. Accelerometry offers an opportunity for remote monitoring which is valuable in tele-health and tele-rehabilitation settings. Identifying biomarkers of stroke recovery will potentially help to triage patients who may require a different approach to stroke rehabilitation. The novel approach of using the whole day to promote activity while reducing sedentary behaviour may be beneficial for many people with stroke, particularly poor responders to rehabilitation poststroke. Personalized intervention using theory-based behaviour change techniques could lead to a paradigm shift in how rehabilitation care is delivered early after stroke. Future research would explore a larger implementation trial of this transformative precision health approach to poststroke rehabilitation.

Role of trainees: This research program will contribute to the training of the next generation of rehabilitation scientists. Trainees will include entry-level physical therapy graduate students, thesis-based rehabilitation science graduate students, and post-doctoral fellows. Graduate students will be trained in quantitative methods of inquiry in stroke research, intervention development, outcome measures, and the use of accelerometry, including data processing and novel compositional data analysis. Current trainees to work on this project include one PhD student and one MSc student. We plan to recruit a postdoctoral fellow by September 2023.

Sex, gender, and other determinants of health in the proposed project?

There are sex differences in stroke outcomes and we will take this into consideration.[53,54] In a pooled analysis of 19,652 survivors of stroke (40% females), females with ischemic stroke were more likely to have poorer global disability (odds ratio for modified Rankin Scale score ≥3 was 1.20 [95% CI: 1.01 to 1.34]).[54] Females and males will be

recruited for this project and effort will be made to have a balanced number of males and females. We will include sex in the generalized estimating equations to account for differences in activity behaviour, mobility, and rehabilitation outcomes. It is anticipated that the biological and socio-cultural identity factors will be similar across intervention and control groups, minimizing bias. We will confirm this by examining baseline differences between intervention and control groups and adjust statistical models if required. Data collection will include biological sex and socio-cultural factors (age, marital status, race/ethnicity, education, and socioeconomic status). We will test sex differences in the primary outcomes (TUG test and mRS).

Time commitment to participate: The measurements will take about 50 minutes per time period, in addition to about 1 hour MRI acquisition/processing time (only at baseline) as outlined.

Outcome measures:

Modified Rankin Scale: (2 minutes)

Timed-Up and Go test: (3 minutes)

ActivPAL set up: (10 minutes)

Ten meter walk test: (5 minutes)

Six minute walk test: (10 minutes)

Montreal Cognitive Assessment (10 minutes)

Euro-QoL 5D (5 minutes)

National Institute of Health Stroke Scale (6 minutes)

MRI acquisition/processing (at baseline only, 60 minutes)

BIG STEPS **intervention:** 1 hour/day, 5x/week, for 6 weeks: 30 hours

Total = 2.5 hours (measurements) + 30 hours (intervention) = **32.5 hours**

Team members:

Project Lead: Victor Ezeugwu, PT, PhD

Principal Investigators:

Brian Buck, MD

Glen Jickling, MD

Study Coordinator/Graduate Students/Research Assistants:

Paige Fairall

Joy Ezeugwa

Aiza Khan

References

1. Wondergem R, Pisters MF, Wouters EJ, de Bie RA, Veenhof C, Visser-Meily JM. The course of physical functioning in the first two years after stroke depends on peoples' individual movement behavior patterns. *Int J Stroke*. 2022;17:83-92. doi: 10.1177/17474930211006293
2. Wardlaw JM, Smith EE, Biessels GJ, Cordonnier C, Fazekas F, Frayne R, Lindley RI, O'Brien JT, Barkhof F, Benavente OR, et al. Neuroimaging standards for research into small vessel disease and its contribution to ageing and neurodegeneration. *Lancet Neurol*. 2013;12:822-838. doi: 10.1016/S1474-4422(13)70124-8
3. Benson J, Seyedsaadat SM, Mark I, Nasr DM, Rabinstein AA, Kallmes DF, Brinjikji W. Leukoaraiosis and acute ischemic stroke: 90-day clinical outcome following endovascular recanalization, with proposed “L-ASPECTS”. *J Neurointerv Surg*. 2021;13:384-389. doi: 10.1136/neurintsurg-2020-015957
4. Helenius J, Henninger N. Leukoaraiosis Burden Significantly Modulates the Association Between Infarct Volume and National Institutes of Health Stroke Scale in Ischemic Stroke. *Stroke*. 2015;46:1857-1863. doi: 10.1161/STROKEAHA.115.009258
5. Taraldsen K, Askim T, Sletvold O, Einarsen EK, Grüner Bjåstad K, Indredavik B, Helbostad JL. Evaluation of a Body-Worn Sensor System to Measure Physical Activity in Older People With Impaired Function. *Phys Ther*. 2011;91:277-285. doi: 10.2522/ptj.20100159
6. Conforto AB, Liew SL, Luft AR, Kitago T, Bernhardt J, Arenillas JF. Editorial: Understanding stroke recovery to improve outcomes: From acute care to chronic rehabilitation. *Front Neurol*. 2022;13:1021033. doi: 10.3389/fneur.2022.1021033
7. Senda J, Ito K, Kotake T, Kanamori M, Kishimoto H, Kadono I, Suzuki Y, Katsuno M, Nishida Y, Ishiguro N, et al. Association of Leukoaraiosis With Convalescent Rehabilitation Outcome in Patients With Ischemic Stroke. *Stroke*. 2016;47:160-166. doi: 10.1161/STROKEAHA.115.010682
8. Liew SL, Zavaliangos-Petropulu A, Jahanshad N, Lang CE, Hayward KS, Lohse KR, Juliano JM, Assogna F, Baugh LA, Bhattacharya AK, et al. The ENIGMA Stroke Recovery Working Group: Big data neuroimaging to study brain-behavior relationships after stroke. *Hum Brain Mapp*. 2022;43:129-148. doi: 10.1002/hbm.25015
9. Stinear CM, Smith M-C, Byblow WD. Prediction Tools for Stroke Rehabilitation. *Stroke*. 2019;50:3314-3322. doi: 10.1161/STROKEAHA.119.025696
10. Wade DT. Rehabilitation potential: A critical review of its meaning and validity. *Clin Rehabil*. 2022:2692155221147606. doi: 10.1177/02692155221147606
11. Regenhardt RW, Takase H, Lo EH, Lin DJ. Translating concepts of neural repair after stroke: Structural and functional targets for recovery. *Restor Neurol Neurosci*. 2020;38:67-92. doi: 10.3233/RNN-190978
12. Prabhakaran S, Zarahn E, Riley C, Speizer A, Chong JY, Lazar RM, Marshall RS, Krakauer JW. Inter-individual Variability in the Capacity for Motor Recovery After Ischemic Stroke. *Neurorehabilitation and Neural Repair*. 2008;22:64-71. doi: 10.1177/1545968307305302
13. Hankey GJ, Spiesser J, Hakimi Z, Bego G, Carita P, Gabriel S. Rate, degree, and predictors of recovery from disability following ischemic stroke. *Neurology*. 2007;68:1583-1587. doi: 10.1212/01.wnl.0000260967.77422.97
14. Hordacre B, Austin D, Brown KE, Graetz L, Parees I, De Trane S, Vallence AM, Koblar S, Kleinig T, McDonnell MN, et al. Evidence for a Window of Enhanced Plasticity in the Human Motor Cortex Following Ischemic Stroke. *Neurorehabil Neural Repair*. 2021;35:307-320. doi: 10.1177/1545968321992330
15. Dromerick AW, Geed S, Barth J, Brady K, Giannetti ML, Mitchell A, Edwardson MA, Tan MT, Zhou Y, Newport EL, et al. Critical Period After Stroke Study (CPASS): A phase II

clinical trial testing an optimal time for motor recovery after stroke in humans. *Proc Natl Acad Sci U S A*. 2021;118. doi: 10.1073/pnas.2026676118
16. Jeffers MS, Karthikeyan S, Corbett D. Does Stroke Rehabilitation Really Matter? Part A: Proportional Stroke Recovery in the Rat. *Neurorehabil Neural Repair*. 2018;32:3-6. doi: 10.1177/1545968317751210
17. Bernhardt J, Churilov L, Ellery F, Collier J, Chamberlain J, Langhorne P, Lindley RI, Moodie M, Dewey H, Thrift AG, et al. Prespecified dose-response analysis for A Very Early Rehabilitation Trial (AVERT). *Neurology*. 2016;86:2138-2145. doi: 10.1212/WNL.0000000000002459
18. Huo L, Chen P, Wang Z, Li X, Zhou J, Wang C, Xing D, Wang S. Impact of leukoaraiosis severity on the association of outcomes of mechanical thrombectomy for acute ischemic stroke: a systematic review and a meta-analysis. *J Neurol*. 2021;268:4108-4116. doi: 10.1007/s00415-020-10167-0
19. Grosu S, Lorbeer R, Hartmann F, Rospleszcz S, Bamberg F, Schlett CL, Galie F, Selder S, Auweter S, Heier M, et al. White matter hyperintensity volume in pre-diabetes, diabetes and normoglycemia. *BMJ Open Diabetes Res Care*. 2021;9:e002050-e002050. doi: 10.1136/bmjdrc-2020-002050
20. Debette S, Seshadri S, Beiser A, Au R, Himali JJ, Palumbo C, Wolf PA, DeCarli C. Midlife vascular risk factor exposure accelerates structural brain aging and cognitive decline. *Neurology*. 2011;77:461-468. doi: 10.1212/WNL.0b013e318227b227
21. Power MC, Deal JA, Sharrett AR, Jack CR, Knopman D, Mosley TH, Gottesman RF. Smoking and white matter hyperintensity progression: The ARIC-MRI Study. *Neurology*. 2015;84:841-848. doi: 10.1212/WNL.0000000000001283
22. Callisaya ML, Beare R, Phan TG, Blizzard L, Thrift AG, Chen J, Srikanth VK. Brain Structural Change and Gait Decline: A Longitudinal Population-Based Study. *Journal of the American Geriatrics Society*. 2013;61:1074-1079. doi: 10.1111/jgs.12331
23. Pinter D, Ritchie SJ, Doubal F, Gattringer T, Morris Z, Bastin ME, del C. Valdés Hernández M, Royle NA, Corley J, Muñoz Maniega S, et al. Impact of small vessel disease in the brain on gait and balance. *Sci Rep*. 2017;7:41637-41637. doi: 10.1038/srep41637
24. Zhang J, Puri AS, Khan MA, Goddeau RP, Henninger N. Leukoaraiosis Predicts a Poor 90-Day Outcome after Endovascular Stroke Therapy. *Am J Neuroradiol* 2014;35:2070-2075. doi: 10.3174/ajnr.A4029
25. Appleton JP, Woodhouse LJ, Adami A, Becker JL, Berge E, Cala LA, Casado AM, Caso V, Christensen HK, Dineen RA, et al. Imaging markers of small vessel disease and brain frailty, and outcomes in acute stroke. *Neurology*. 2020;94:e439-e452. doi: 10.1212/WNL.0000000000008881
26. Braun RG, Heitsch L, Cole JW, Lindgren AG, de Havenon A, Dude JA, Lohse KR, Cramer SC, Worrall BB. Domain-Specific Outcomes for Stroke Clinical Trials. *Neurology*. 2021;97:367-377. doi: 10.1212/WNL.0000000000012231
27. Weisscher N, Vermeulen M, Roos YB, Haan RJ. What should be defined as good outcome in stroke trials; a modified Rankin score of 0–1 or 0–2? *J Neurol*. 2008;255:867-874. doi: 10.1007/s00415-008-0796-8
28. Erler KS, Wu R, DiCarlo JA, Petrilli MF, Gochyyev P, Hochberg LR, Kautz SA, Schwamm LH, Cramer SC, Finklestein SP, et al. Association of Modified Rankin Scale With Recovery Phenotypes in Patients With Upper Extremity Weakness After Stroke. *Neurology*. 2022;98:e1877-e1885. doi: 10.1212/WNL.0000000000200154
29. Ganesh A, Ospel JM, Menon BK, Demchuk AM, McTaggart RA, Nogueira RG, Poppe AY, Almekhlafi MA, Hanel RA, Thomalla G, et al. Assessment of Discrepancies Between Follow-up Infarct Volume and 90-Day Outcomes Among Patients With Ischemic Stroke

Who Received Endovascular Therapy. *JAMA Netw Open*. 2021;4:e2132376-e2132376. doi: 10.1001/jamanetworkopen.2021.32376
30. Dumuid D, Pedišić Ž, Palarea-Albaladejo J, Martín-Fernández JA, Hron K, Olds T. Compositional Data Analysis in Time-Use Epidemiology: What, Why, How. *Int J Environ Res Public Health*. 2020;17:2220-2220. doi: 10.3390/ijerph17072220
31. Dumuid D, Olds T, Sawyer SM. Moving beyond more: towards a healthy balance of daily behaviours. *Lancet*. 2021;398:373-374. doi: 10.1016/S0140-6736(21)01600-7
32. Chastin SF, Palarea-Albaladejo J, Dontje ML, Skelton DA. Combined Effects of Time Spent in Physical Activity, Sedentary Behaviors and Sleep on Obesity and Cardio-Metabolic Health Markers: A Novel Compositional Data Analysis Approach. *PLoS One*. 2015;10:e0139984. doi: 10.1371/journal.pone.0139984
33. Li J, Cao D, Huang Y, Chen Z, Wang R, Dong Q, Wei Q, Liu L. Sleep duration and health outcomes: an umbrella review. *Sleep Breath*. 2022;26:1479-1501. doi: 10.1007/s11325-021-02458-1
34. Ezeugwu VE, Manns PJ. The Feasibility and Longitudinal Effects of a Home-Based Sedentary Behavior Change Intervention After Stroke. *Arch Phys Med Rehabil*. 2018;99:2540-2547. doi: 10.1016/j.apmr.2018.06.014
35. Ezeugwu VE, Manns PJ. Using Intervention Mapping to develop and implement a home-based sedentary behavior change intervention after stroke. *Transl Behav Med*. 2020;10:87-95. doi: 10.1093/tbm/iby128
36. Klassen TD, Semrau JA, Dukelow SP, Bayley MT, Hill MD, Eng JJ. Consumer-Based Physical Activity Monitor as a Practical Way to Measure Walking Intensity During Inpatient Stroke Rehabilitation. *Stroke*. 2017;48:2614-2617. doi: 10.1161/STROKEAHA.117.018175
37. Podsiadlo D, Richardson S. The Timed “Up & Go”: A Test of Basic Functional Mobility for Frail Elderly Persons. *Journal of the American Geriatrics Society*. 1991;39:142-148. doi: 10.1111/j.1532-5415.1991.tb01616.x
38. Simpson DB, Breslin M, Cumming T, de Zoete SA, Gall SL, Schmidt M, English C, Callisaya ML. Sedentary time and activity behaviors after stroke rehabilitation: Changes in the first 3 months home. *Top Stroke Rehabil*. 2021;28:42-51. doi: 10.1080/10749357.2020.1783917
39. Fulk GD, Echternach JL. Test-Retest Reliability and Minimal Detectable Change of Gait Speed in Individuals Undergoing Rehabilitation After Stroke. *J Neurol Phys Ther*. 2008;32:8-13. doi: 10.1097/NPT0b013e31816593c0
40. Kwakkel G, Lannin NA, Borschmann K, English C, Ali M, Churilov L, Saposnik G, Winstein C, van Wegen EEH, Wolf SL, et al. Standardized Measurement of Sensorimotor Recovery in Stroke Trials: Consensus-Based Core Recommendations from the Stroke Recovery and Rehabilitation Roundtable. *Neurorehabil Neural Repair*. 2017;31:784-792. doi: 10.1177/1545968317732662
41. Fulk GD, Echternach JL, Nof L, O'Sullivan S. Clinometric properties of the six-minute walk test in individuals undergoing rehabilitation poststroke. *Physiother Theory Pract*. 2008;24:195-204. doi: 10.1080/09593980701588284
42. Zietemann V, Georgakis MK, Dondaine T, Müller C, Mendyk A-M, Kopczak A, Hénon H, Bombois S, Wollenweber FA, Bordet R, et al. Early MoCA predicts long-term cognitive and functional outcome and mortality after stroke. *Neurology*. 2018;91:e1838-e1850. doi: 10.1212/WNL.0000000000006506
43. Golicki D, Niewada M, Buczek J, Karlińska A, Kobayashi A, Janssen MF, Pickard AS. Validity of EQ-5D-5L in stroke. *Qual Life Res*. 2015;24:845-850. doi: 10.1007/s11136-014-0834-1

44. Xie F, Pullenayegum E, Gaebel K, Bansback N, Bryan S, Ohinmaa A, Poissant L, Johnson JA. A Time Trade-off-derived Value Set of the EQ-5D-5L for Canada. *Med Care*. 2016;54:98-105. doi: 10.1097/MLR.0000000000000447
45. Jickling GC, Ander BP, Zhan X, Stamova B, Hull H, DeCarli C, Sharp FR. Progression of cerebral white matter hyperintensities is related to leucocyte gene expression. *Brain*. 2022;145:3179-3186. doi: 10.1093/brain/awac107
46. Hammam N, Sadeghi D, Carson V, Tamana SK, Ezeugwu VE, Chikuma J, Van Eeden C, Brook JR, Lefebvre DL, Moraes TJ, et al. The relationship between machine-learning-derived sleep parameters and behavior problems in 3- and 5-year-old children: Results from the CHILD Cohort study. *Sleep*. 2020;43:zsaa117-zsaa117. doi: 10.1093/sleep/zsaa117
47. Mannini A, Trojaniello D, Cereatti A, Sabatini AM. A Machine Learning Framework for Gait Classification Using Inertial Sensors: Application to Elderly, Post-Stroke and Huntington's Disease Patients. *Sensors (Basel)*. 2016;16. doi: 10.3390/s16010134
48. Boyd LA, Hayward KS, Ward NS, Stinear CM, Rosso C, Fisher RJ, Carter AR, Leff AP, Copland DA, Carey LM, et al. Biomarkers of stroke recovery: Consensus-based core recommendations from the Stroke Recovery and Rehabilitation Roundtable. *Int J Stroke*. **2017;12:480-493. doi: 10.1177/1747493017714176**
49. **Katan M, Luft A. Global Burden of Stroke. *Semin Neurol*. 2018;38:208-211. doi: 10.1055/s-0038-1649503**
50. Ezeugwu VE, Manns PJ. Sleep Duration, Sedentary Behavior, Physical Activity, and Quality of Life after Inpatient Stroke Rehabilitation. *J Stroke Cerebrovasc Dis*. 2017;26:2004-2012. doi: 10.1016/j.jstrokecerebrovasdis.2017.06.009
51. Barrett M, Snow JC, Kirkland MC, Kelly LP, Gehue M, Downer MB, McCarthy J, Ploughman M. Excessive sedentary time during in-patient stroke rehabilitation. *Top Stroke Rehabil*. 2018;25:366-374. doi: 10.1080/10749357.2018.1458461
52. Wang Z, Jin X, Liu Y, Wang C, Li J, Tian L, Teng W. Sedentary behavior and the risk of stroke: A systematic review and dose-response meta-analysis. *Nutr Metab Cardiovasc Dis*. 2022;32:2705-2713. doi: 10.1016/j.numecd.2022.08.024
53. Tanlaka E, King-Shier K, Green T, Seneviratne C, Dukelow S. Sex Differences in Stroke Rehabilitation Care in Alberta. *Can J Neurol Sci*. 2020;47:494-503. doi: 10.1017/cjn.2020.53
54. Carcel C, Wang X, Sandset EC, Delcourt C, Arima H, Lindley R, Hackett ML, Lavados P, Robinson TG, Munoz Venturelli P, et al. Sex differences in treatment and outcome after stroke: Pooled analysis including 19,000 participants. *Neurology*. 2019;93:e2170-e2180. doi: 10.1212/WNL.0000000000008615

Figure 1: Timed-up and-go (TUG) score adjusted for age, sex, and cognition: Predicted change in TUG scores with an increase in time spent in one behaviour relative to a proportional decrease in the other behaviours

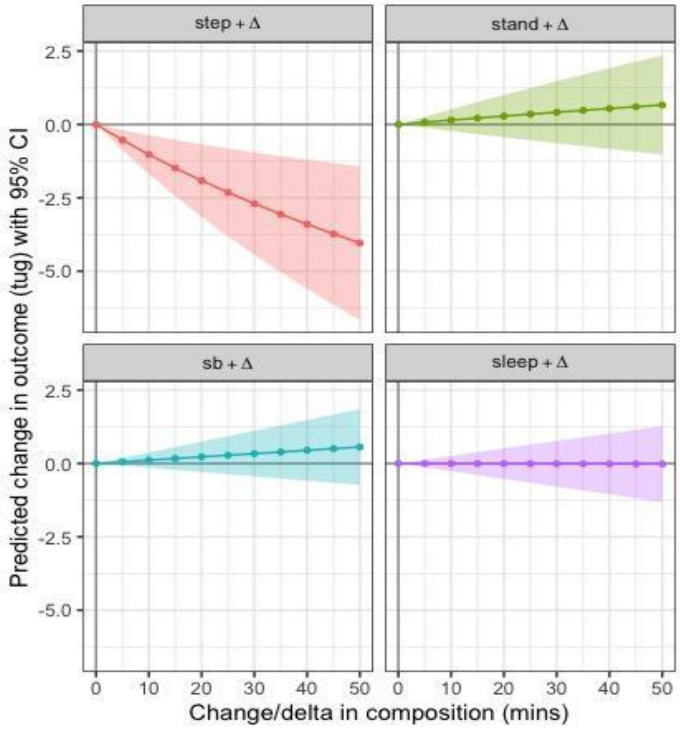

Notes: step=stepping, stand =standing, sb = sedentary behaviour
Tail: represents 95% confidence interval
